# High-efficiency promoter-driven coordinated regulation of multiple metabolic nodes elevates lipid accumulation in the model microalga *Phaeodactylum tricornutum*

**DOI:** 10.1186/s12934-018-0906-y

**Published:** 2018-04-04

**Authors:** Li-Gong Zou, Jia-Wen Chen, Dan-Lin Zheng, Srinivasan Balamurugan, Da-Wei Li, Wei-Dong Yang, Jie-Sheng Liu, Hong-Ye Li

**Affiliations:** Key Laboratory of Eutrophication and Red Tide Prevention of Guangdong Higher Education Institutes, College of Life Science and Technology, Jinan University, Guangzhou, 510632 China

**Keywords:** Microalga, Promoter, Triacylglycerol, Metabolic nodes

## Abstract

**Background:**

Microalgal metabolic engineering holds great promise for the overproduction of a wide range of commercial bioproducts. It demands simultaneous manipulation of multiple metabolic nodes. However, high-efficiency promoters have been lacking.

**Results:**

Here we report a strong constitutive promoter Pt211 in expressing multiple target genes in oleaginous microalga *Phaeodactylum tricornutum*. Pt211 was revealed to contain significant *cis*-acting elements. GUS reporter and principal genes glycerol-3-phosphate acyltransferase (GPAT) and diacylglycerol acyltransferase 2 (DGAT2) involved in triacylglycerol biosynthesis were tested under driven of Pt211 in *P. tricornutum.* GUS staining and qPCR analysis showed strong GUS expression. DGAT2 and GPAT linked with a designed 2A sequence exhibited higher transcript abundances than WT, while algal growth and photosynthesis were not impaired.

**Conclusion:**

The total lipid content increased notably by 2.6-fold compared to WT and reached up to 57.5% (dry cell weight). Overall, our findings report a strong promoter and a strategy for coordinated manipulation of complex metabolic pathways.

## Background

Microalgae are of significant interest as renewable feedstock for biofuels and a wide range of commodity components because of their advantageous features such as phototrophic nature, high growth rate, oleaginous biomass and importantly, they do not compete with food crops for arable land [1, 2]. Oleaginous marine diatom *Phaeodactylum tricornutum* have emerged as a popular model system for molecular characterization of metabolism [3]. In addition, *P. tricornutum* has been served as the potential host for expression of various recombinant therapeutic proteins [4, 5]. Unlike stress treatments, genetic engineering of diatoms paves the way for lipid overproduction without compromising the growth potential and thus considered a pivotal strategy for mitigating economic feasibility [6]. Genetic engineering of *P. tricornutum* for lipid overproduction needs the controlled expression of multiple key genes involved in lipogenesis. Thus, the transcriptional regulation of the desired genes plays a crucial role in the transgene expression, which is largely regulated by promoters and their *cis*-acting elements.

Most of the genetic engineering studies have employed the promoters such as fucoxanthin chlorophyll a/c binding protein (fcp) promoter and nitrate reductase (NR) promoter in microalgae including *P. tricornutum* [6, 7]. The fcp and NR promoters isolated from diatom *Cylindrotheca fusiformis* showed effective in *P. tricornutum* [8]. The NR promoter isolated from *P. tricornutum* has also been shown useful for transgene regulation [6]. Similarly, *fcpC* promoter from *P. tricornutum* has been shown to regulate heterologous protein expression in *C. pyrenoidosa* [9]. However, to date, only a limited number of promoters have been employed for successful driving transgene expression in *P. tricornutum*. Although significant progresses have been made regarding heterologous expression in *P. tricornutum* in recent years [1, 10], the promoters that strongly drive the transcription of multiple transgenes have yet to be discovered for expanding the microalgal genetic toolbox.

In this study, we aimed to identify the strong promoter suitable for initiating the transcription of multi-transgene in *P. tricornutum*. Based on our previous transcriptomic analysis [11], promoter Pt211 was identified and further evaluated in efficiently driving multiple target genes, including GUS reporter and two principle genes involved in TAG biosynthesis.

## Methods

### Strain and culture conditions

*Phaeodactylum tricornutum* (CCMP2561) was obtained from the National Center for Marine Algae and Microbiota. It was cultivated in Daigo’s IMK culture medium (Nihon Pharmaceutical, Osaka, Japan) [12] and grown at 21 ± 1 °C in an artificial climate incubator (Ningbo, China). Cool-white fluorescent tubes provided an irradiance of 200 µmol photons m^−2^ s^−1^ under a 12:12 h light/dark photoperiod.

### Identification of promoter sequence and vector construction

Based on our previously analyzed transcriptome of *P. tricornutum* [11], the upstream region to the hypothetical protein gene (PHATRDRAFT_49211) with higher transcript abundance was selected. The potential cis regulatory elements were predicted by using PlantCARE (http://bioinformatics.psb.ugent.be/webtools/plantcare/html) [13, 14]. Genomic DNA of *P. tricornutum* was extracted using HP Plant DNA Kit as per supplier’s protocol (OMEGA, USA). The putative promoter region upstream to the gene PHATRDRAFT_49211 was designated Pt211 and cloned by PCR with primers (211-F and 211-R). In the transformation construct pHY21 was modified from a previously constructed plasmid, pHY11 [12]. The amplicon was then cloned in pHY21 expression vector to replace the existing promoter fcpC by using ClonExpress II One Step Cloning Kit (Vazyme, China) and the resultant recombinant plasmid was termed pHY21-Pt211 and confirmed by sequencing analysis. Thereafter, coding regions of reporter GUS, or DGAT2 and GPAT were linked with a codon-optimized 2A sequence (5′-AAGATCGTCGCCCCCGTCAAGCAGACCCTCAACTTCGACCTCCTCAAGCTCGCCGGCGACGTCGAGTCCAACCCCGGCCCC-3′). Flag and c-Myc tags were fused to the C-terminal of the transgenes *DGAT2* and *GPAT*, respectively in the expression vector for the detection of protein expression. The coding sequences were cloned in between the identified promoter *Pt211* and *TfcpA* terminator in the expression vector pHY21. An omega leader motif (TATTTTTACAACAATTACCAACAACAACAAACAACAAACAACATTACAATTACTATTTACAATT) was inserted in between the promoter and the transgene in order to enhance the translation of transgene [15]. In addition, DGAT2 and GPAT linked with 2A were also cloned in pHY21 with the promoter fcpC for comparison.

The recombinant plasmid harboring GUS and DGAT2-2A-GPAT under the control of Pt211 were electroporated into *P. tricornutum* using Bio-Rad GenePulser Xcell apparatus (Bio-Rad, CA, USA) following the reported protocol (Table 1) [3]. Transformed microalgae were subcultured once a week. To avoid impact of bleomycin on microalgae, engineered strains were cultured in IMK culture medium without bleomycin for more than 3 successive subculture cycles before biochemical and physiological measurements. Growth curve was determined in triplicates using a brightline hemocytometer under light microscope every day.

**Table 1:** Plasmids and strains used in this work

Strains	Plasmid	Source
	pHY11	Niu et al. [12]
	pHY21: *PfcpC*-*EGFP*- *TfcpA*- *Ble*	This work
Pt211-GUS	*pPt211*-*GUS*	This work
Pt211	*pPt211*-*DGAT2*-Flag-2A-*GPAT*-Myc	This work

### Screening of transformants by molecular approaches

The incorporation of constructed expression cassettes into diatom genome was investigated by colony genomic PCR. After electroporation, the transformants were spread on the culture plates supplemented with bleomycin under standard conditions. After 2–3 weeks, survived colonies were picked and inoculated in 5 ml liquid medium containing bleomycin. In the stationary phase (~ 10 days), 1 ml of the culture was centrifuged and resuspended in 20 µl ddH_2_O. Finally 1 µl was used as the template for a 20 µl-reaction of genomic PCR. Primers Ble-F and Ble-R were used to amplify the integration of *Ble* gene [16]. GUS assay was performed to detect the GUS gene expression. It was performed on cells (corresponding to 7.5 × 10^6^ cells) resuspended in 200 µl GUS extraction buffer [50 mM sodium phosphate (pH 7.0), 10 mM EDTA (pH 8.0), 0.1% SDS, 0.1% Triton X-100, and freshly added 10 mM β-mercaptoethanol and 25 µg ml^−1^ PMSF] and 4 µl X-Gluc, for 12 h at 37 °C [17].

The relative transcript abundance of GUS, DGAT2 and GPAT, were determined by quantitative real-time PCR (qPCR) with qPCR SYBR Green master mix (Vazyme, China) and performed on BIO-RAD CFX96 (BioRad, USA). Total RNA was isolated from microalgae using Plant RNA Kit (OMEGA, USA) and reversely transcribed into first strand cDNA using PrimeScript™ RT Reagent Kit (Takara, Japan). qPCR analysis was performed following the method as previously described [6]. The relative transcript abundance was determined by the 2^−ΔΔCt^ method after normalization to the endogenous control gene β-actin.

To examine the protein expression of DGAT2 and GPAT in transformants, Western blot analysis was performed against anti-c-Myc and anti-Flag tags as described previously. An anti-Flag antibody (1:3500; Sigma-Aldrich, USA) and anti-Myc antibody (1:5000; Sigma-Aldrich, USA) were used as the corresponding primary antibodies, respectively. HRP-conjugated goat anti-rabbit secondary antibody (1:5000, CST, USA) was used as the secondary antibody. The membrane was developed by chemiluminescence system. β-Actin was used as internal reference.

### Analysis of photosynthetic activity

The maximum quantum yield of photosystem II (Fv/Fm; ratio of variable/maximum fluorescence) of microalgae was measured using phytoplankton analyzer (WALZ, Germany).

### Fluorometric and gravimetric analysis of lipid content

Cellular neutral lipid content was determined by using Nile Red staining (Sigma, USA) according to Yang et al. [11]. Staining was performed in a 96-well microtitre plate in triplicates and fluorescence was recorded using Synergy 4 Hybrid (BioTek, USA) at a wavelength of 530 nm excitation and 580 nm emission. For confocal microscopic observation of microalgal morphology, cells were stained with Nile red and lucifugally incubated for 10 min at room temperature. Stained cells were observed under a laser-scanning confocal microscope Zeiss LSM510meta (Zeiss, Germany) with an excitation wavelength of 488 nm and an emission wavelength of 505–550 nm.

Total lipids from the microalgae were extracted as per the protocol reported by Bligh and Dyer [27]. Cells were subjected to nitrogen deprivation to validate the lipid accumulating capability as previously described [9]. Fatty acid composition of the total lipids was analyzed as fatty acid methyl esters (FAMEs) by using gas chromatography–mass spectrometry (GC–MS) according to the protocol reported previously [11].

## Results and discussion

### In silico analysis and molecular cloning of the promoter Pt211

**Fig. 1:**
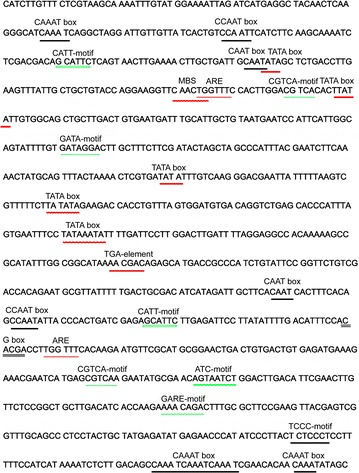
Schematic map of *cis*-acting elements predicted in Pt211. The predicted *cis*-acting regulatory elements are underlined

Based on our previously analyzed transcriptome of *P. tricornutum* [11], the upstream region to the hypothetical protein gene (PHATRDRAFT_49211) with higher transcript abundance was selected. The 1020-bp upstream region designated Pt211 was predicted by using the online tools such as PLACE and PlantCARE and 23 revealed the presence of 19 types of potential *cis*-elements. Interestingly, we found that Pt211 harbors critical *cis*-regulatory elements including 21 TATA boxes and 22 CAAT boxes that are essential in transcription (Fig. 1). Thereafter, the putative promoter Pt211 containing the necessary regulatory elements was subjected to further analysis for its transcriptional regulatory role.

### Experimental validation of Pt211 promoter using GUS reporter

**Fig. 2:**
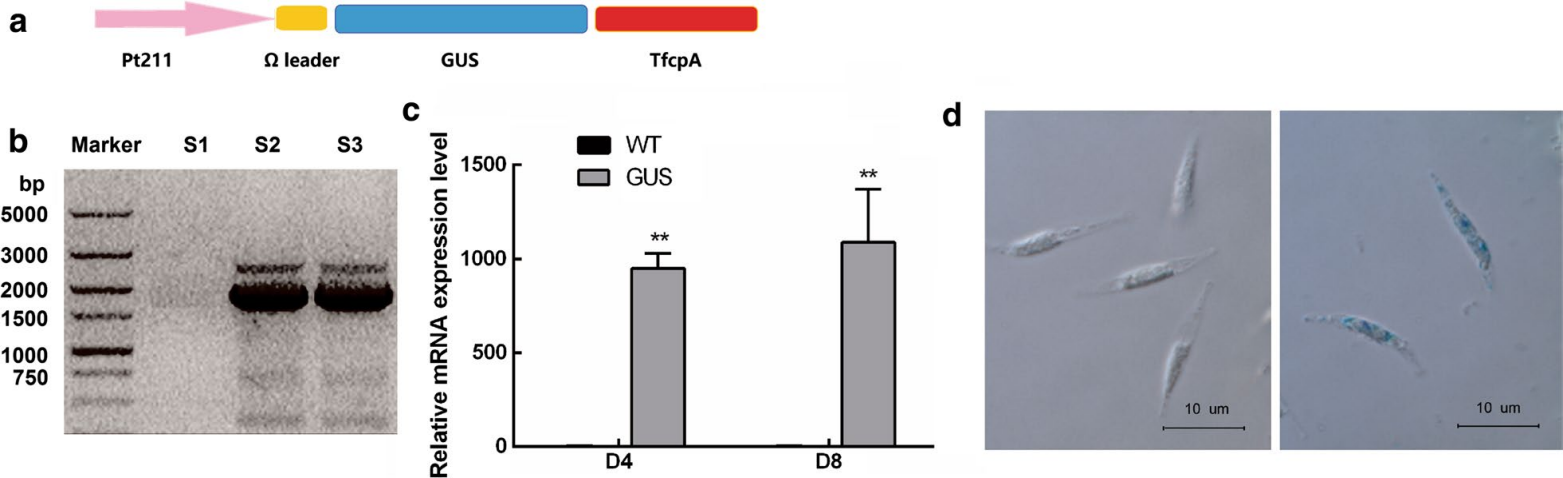
Characterization of Pt211 promoter. **a** Schematic map of the Pt211-GUS expression cassette. **b** Genomic PCR analysis. S1: WT, S2-3: Pt211-GUS-1, Pt211-GUS-2, M: molecular marker. Lane M: 5000 bp DNA ladder. **c** Relative transcript abundance of Pt211 driven *GUS* expression determined by qPCR. D4 and D8 indicate the fourth and 8th day of the cultivation period. ** indicates a significant difference (*p* < 0.01). Each value represents mean ± SD (n = 3). **d** GUS staining of microalgae observed under a fluorescence microscope

To assess the activity of identified Pt211, *GUS* reporter gene was cloned under the control of Pt211 (Fig. 2a) and the *P. tricornutum* was transformed with the recombinant plasmid pPt211-GUS. Molecular analyses including colony genomic PCR and qPCR were further carried out. Genomic PCR analysis revealed the presence of 1.8-kb amplicon *GUS* reporter in engineered microalgae, whereas no such band was present in WT (Fig. 2b). In order to investigate the promoter activity, the relative transcript abundance of *GUS* expression driven by Pt211 was determined. As shown in Fig. 2c, the transcription of *GUS* was detected only in the engineered strains and it was found to be relatively quite high compared to the internal reference actin gene. Subsequently, the GUS staining signal in microalgae was observed under a fluorescence microscope. As shown in Fig. 2d, engineered microalgae exhibited specific GUS staining signal (blue color) whereas WT showed no GUS staining.

### Construction of double gene expression cassette under the control of Pt211 promoter and algal transformation

**Fig. 3:**
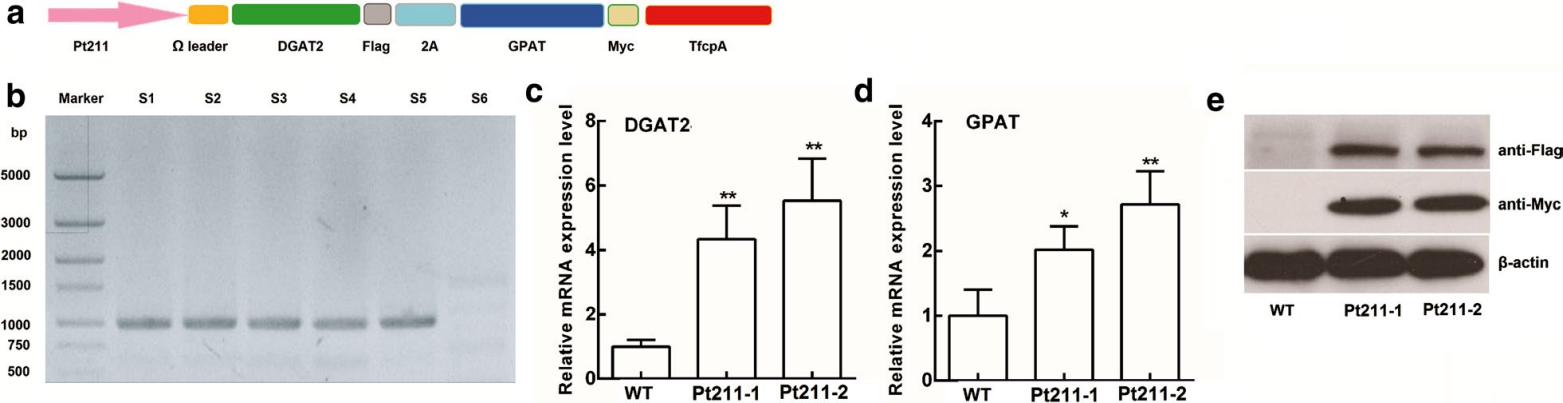
Molecular analysis of engineered microalgae. **a** Schematic representation of the Pt211-D2G (DGAT2 and GPAT) construct. **b** Genomic PCR analysis of engineered strains showed the integration of Pt211-D2G construct. S1–S5: engineered strains, S6: WT, M: molecular marker. **c**, **d** qPCR analysis of DGAT2 (**c**) and GPAT (**d**) transcript levels. * and ** indicates the significant difference at *p* < 0.05 and *p* < 0.01 level, respectively. Each value represents mean ± SD (n = 3). **e** Western blot analysis. The anti-Flag and anti-Myc antibodies were used for the detection of DGAT2 and GPAT, respectively

After successful verification of the driving role of promoter Pt211 using GUS, we then employed Pt211 to drive the expression of DGAT2 and GPAT, the key genes involved in de novo TAG biosynthetic pathway, to test its potential in biotechnology application. An omega leader sequence was added before the coding region for enhancing translation, and DGAT2 and GPAT were linked with a designed 2A sequence (Fig. 3a). The recombinant construct pPt211-DGAT2-2A-GPAT was then introduced into *P. tricornutum.*

### Molecular characterization of engineered strains

Several independent engineered strains were screened in terms of molecular and lipid productivity analysis. For clarity and conciseness of the presentation, only the data of two engineered strains were presented and designated Pt211-1 and Pt211-2. To detect the stable integration of expression cassette into the host genome, colony genomic PCR was performed. As expected, a 1.2-kb amplicon of the expression cassette was detected in engineered strains while absent in WT (Fig. 3b). The relative transcript levels of DGAT2 and GPAT driven by Pt211 were determined by qPCR. Results showed that transcript abundance of DGAT2 and GPAT was remarkably increased by 5.5- and 2.7-fold, respectively, compared to that of WT (Fig. 3c, d). Western blotting was carried out to confirm the protein expression. Specific cross-reacting bands of about 40.4- and 35.0-kDa corresponding to DGAT2 and GPAT, respectively, were observed in the engineered strains, while no band was present in WT (Fig. 3e). These results suggest that Pt211 promoter can significantly drive the expression of multi-stack genes.

### Growth and photosynthetic rate of engineered microalgae

**Fig. 4:**
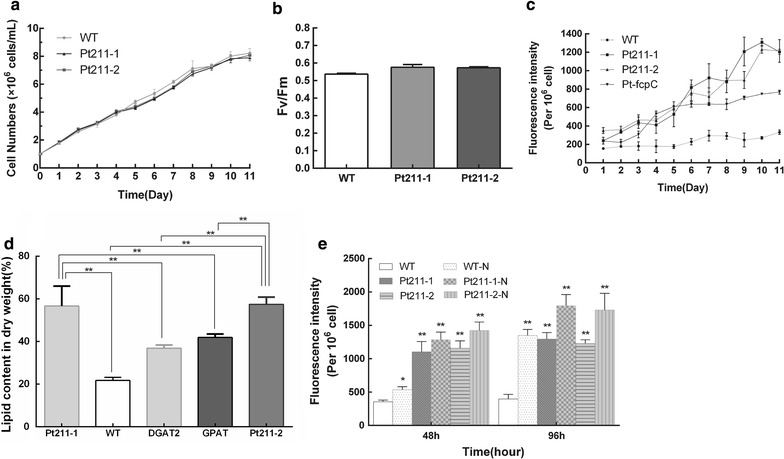
Growth curves and neutral lipid accumulation of microalgae. **a** Growth curve. **b** Photosynthetic efficiency indicated by chlorophyll fluorescence parameter Fv/Fm. **c** Neutral lipid accumulation per culture volume. **d** Neutral lipid accumulation under N deprivation (-N). **e** Total lipid content of microalgal strains determined by gravimetry (dry cell weight). Each value represents mean ± SD (n = 3). * and ** indicate the significant difference at *p* < 0.05 and *p* < 0.01 level, respectively

To evaluate the impact of Pt211 driven DGAT2 and GPAT expression on general physiological and growth characteristics of the cells, growth and photosynthetic performance were determined. As shown in Fig. 4a, both engineered and WT cells exhibited similar growth rates (Fig. 4a). The maximum quantum efficiency of photosystem II (Fv/Fm) was measured to detect the photosynthetic performance and acclimation status. As shown in Fig. 4b, Fv/Fm was slightly higher in engineered strains than WT. These results suggested that Pt211-driven DGAT2 and GPAT overexpression did not impair growth and photosynthetic performance.

### Pt211-driven expression of DGAT2 and GPAT elevated neutral lipid accumulation

Fluorometric and conventional gravimetric analysis of lipids were performed to measure the neutral lipid content in microalgae. As shown in Fig. 4c, fluorometric analysis revealed that the relative neutral lipid content was increased significantly by 3.6- and 3.9-fold in strains Pt211-1 and Pt211-2, respectively, than WT, and also higher than that of conventional fcpC-driven engineered microalgae. Gravimetric analysis of lipid content showed that total lipid content was increased notably by 2.6-fold in engineered strains than WT and reached 56.6 and 57.5% (dry cell weight) in strains Pt211-1 and Pt211-2, respectively. Compared to the DGAT2- or GPAT-overexpressing microalgae [3, 16] previously generated in our lab, both strains Pt211-1 and Pt211-2 exhibited significant increase in total lipid content (Fig. 4d).

It is well established that nitrogen deprivation can stimulate lipid accumulation in microalgae. Thus, the engineered strains were further subjected to nitrogen deprivation in order to achieve the maximum lipid productivity. As shown in Fig. 4e, under -N, both the engineered and WT accumulated more lipid content as expected, while the lipid content was even higher under -N for 96 h than 48 h. Engineered cells showed 1.38-fold higher lipid content under -N for 96 h than that cultured in N-replete medium. Taken together, these results revealed the potential role of Pt211 in governing the gene expression. Taken into account that neutral lipid content was significantly increased in Pt211 driven engineered strains than fcpC driven strains, this impact on lipid increment in Pt211 strains could be explained by a superior transcriptional regulatory role of the identified Pt211 promoter.

### Confocal microscopic examination of algal morphology

**Fig. 5:**
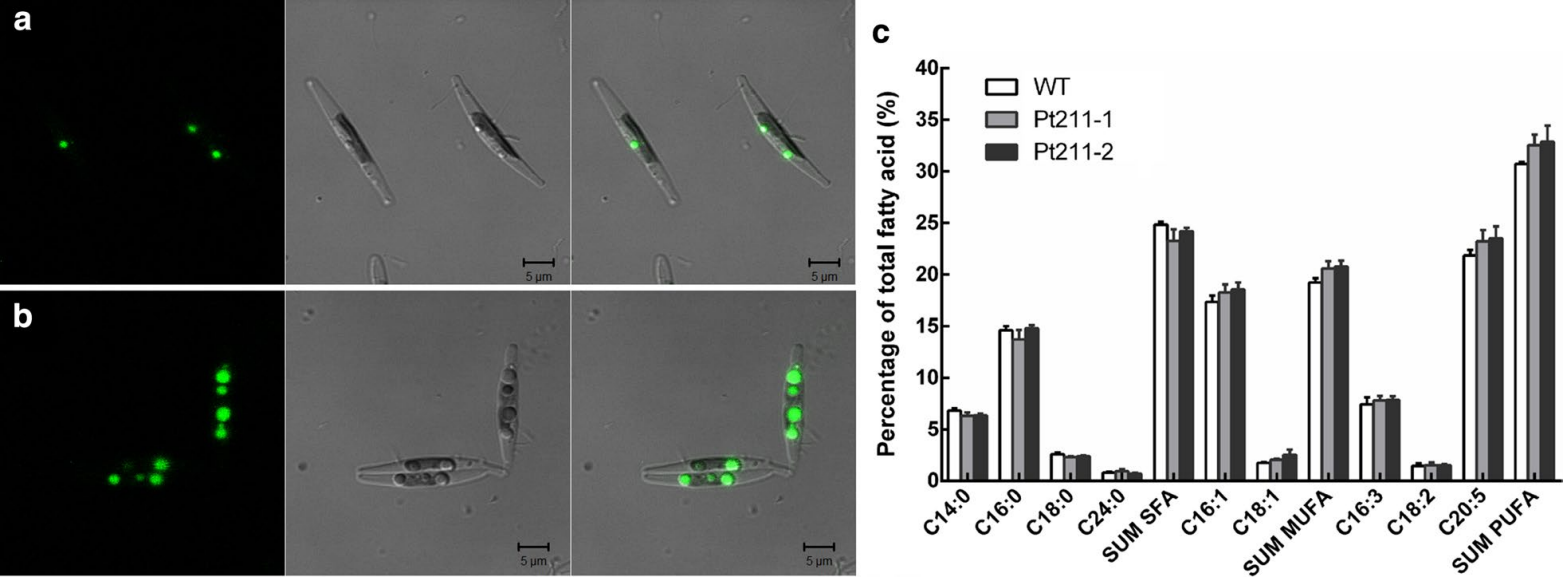
Cell morphology and fatty acid composition. **a** WT. **b** Engineered cells. Left: red fluorescence of neutral lipids; middle: DIC (differential interference contrast); right: overlay image of red fluorescence and DIC. Bar = 5 μm. **c** The relative abundance of fatty acid composition within neutral lipids

To further examine the morphological changes in strains, Nile red stained cells were observed under laser scanning confocal microscope. As shown in Fig. 5a, b, no change was observed in terms of cellular morphology and size between engineered and WT cells. By contrast, the volume and number of lipid droplets were increased concomitantly. This observation indicates that Pt211-driven expression of DGAT2 and GPAT lead to lipid overproduction, which is in accordance with the fluorometric and gravimetric analysis of lipids.

### Impact of Pt211 controlled DGAT2 and GPAT on fatty acid biosynthesis

The effects of DGAT2 and GPAT overexpression on fatty acid composition were investigated in microalgae during the stationary phase by GC–MS analysis. As shown in Fig. 5c, fatty acid composition was altered in engineered strains. Total saturated fatty acid content was decreased in engineered strains. On the other hand, total content of monounsaturated and polyunsaturated fatty acid was found to be increased. Monounsaturated fatty acids are predominantly composed of C16:1 and C18:1, and C16:1 content was even at least 9.0-fold higher than C18:1 content in both engineered and WT cells. Among polyunsaturated fatty acid content, C20:5 as the majority component was increased, while no significant change was observed in terms of C16:3 and C18:2.

## Discussion

Our findings revealed a strong promoter Pt211 which showed higher efficiency in driving multiple gene expression compared to the commonly used promoter fcpC. Metabolic engineering strategies require precisely controlled expression of desired genes in order to achieve the overproduction, as the metabolic flux is controlled by series of reactions and regulatory components during various stages [18]. Lack of high efficiency promoters has hampered the exploitation of microalgae.

Our previous transcriptomic analyses of *P. tricornutum* indicated the presence of putative promoters to drive high transcription. In the present study, we identified the promoter Pt211 and the occurrence of essential regulatory components. In silico analyses revealed the presence of 19 potential *cis*-acting elements in 1.02-kb Pt211 promoter including 21 TATA- and 22 CAAT-boxes. The TATA box has been considered a crucial element in determining the transcription initiation site and also for the recognition of transcription site by transcription factors [19], The CAAT box was known to regulate the transcription frequency [20]. In addition to core *cis* elements, a number of other *cis*-acting regulatory elements were identified in Pt211. These included several light responsive elements (I-box, -84; GATA-motif, -710; Sp1, -447 and TCCC-motif, -71; G-box, -301), phytohormone responsive elements, methyl jasmonate responsive elements and regulatory element associated to the TGAGTCA motif. Its capability of driving exogenous gene was experimentally validated. Our qPCR data revealed that relative transcript abundance of *GUS* reporter was found to be significantly higher in Pt211-GUS strains, which also remained constitutively stable during the whole culture period. GUS histochemical analysis revealed that Pt211 conferred high level GUS activity.

Regulation of multiple genes has been considered to be crucial in metabolic rewiring approaches. Particularly, metabolic engineering for lipid overproduction often necessitates the expression of multiple crucial genes under the control of a strong promoter [21]. Conventional approaches such as sequential transformation with multiple genes had the drawbacks such as labor-intensive, increased copy number of transgenes, etc. [22]. Hence, we attempted and successfully demonstrated the strength of Pt211 promoter in driving multiple genes by fusing two crucial lipid synthesis-associated genes GPAT and DGAT2. Molecular characterization of engineered strains revealed that relative transcript abundance of both GPAT and DGAT2 increased significantly. Lipid accumulation capability was higher than that under control of conventional fcpC promoter. Wang et al. [23] reported that the identified promoter gpd3 was found to be more effective than the routinely used nmt1 promoter in yeast *Schizosaccharomyces pombe* [23]. In order to further evaluate whether the promoter activity was impaired by the nutrient stress, engineered microalgae were subjected to nitrogen deprivation treatment. Importantly, the relative lipid content was remarkably increased in engineered strains subjected to -N than that grown in normal conditions. It has been reported that promoter activity was significantly affected by the environmental factors [24, 25] and chemical components [26]. Intriguingly, our results indicated that promoter Pt211 constitutively expressed multiple genes and the activity was not impaired by nitrogen deprivation.

## Conclusion

A constitutive high efficiency promoter Pt211 with significant c*is*-acting elements was identified, and simultaneous multiple gene expression with the strategy of 2A linker was first succeeded in microalgae. Pt211 driven GPAT-DGAT2 expression resulted in remarkable elevation of relative content and conferred stable expression in engineered microalgae. Overall, the findings provide a new molecular tool kit for coordinated expression of multiple metabolic nodes.

## Abbreviations

ACCase: acetyl-CoA carboxylase
GC–MS: gas chromatography–mass spectrometry
MUFA: monounsaturated fatty acid
PUFA: polyunsaturated fatty acid
qPCR: quantitative polymerase chain reaction
